# Deep Brain Stimulation Combined with NMDA Antagonist Therapy in the Treatment of Alzheimer’s Disease: In Silico Trials

**DOI:** 10.3390/jcm13247759

**Published:** 2024-12-19

**Authors:** Dariusz Świetlik

**Affiliations:** Division of Biostatistics and Neural Networks, Medical University of Gdansk, Debinki 1 St., 80-211 Gdansk, Poland; dariusz.swietlik@gumed.edu.pl

**Keywords:** NMDA antagonists, memantine, Alzheimer’s disease, neural networks, computer simulation, virtual therapy, in silico trials, deep brain stimulation (DBS)

## Abstract

**Background**: Deep brain stimulation (DBS) is employed to adjust the activity of impaired brain circuits. The variability in clinical trial outcomes for treating Alzheimer’s disease with memantine is not yet fully understood. We conducted a randomized in silico study comparing virtual DBS therapies with treatment involving an NMDA antagonist combined with DBS in patients with Alzheimer’s disease. **Methods**: Neural network models representing Alzheimer’s disease (AD) patients were randomly assigned to four groups: AD, memantine treatment, DBS, and DBS and memantine. Out of 100 unique neural networks created to model moderate and severe AD with varying hippocampal synaptic loss, 20 were randomly selected to represent AD patients. Virtual treatments—memantine, DBS, and DBS and memantine—were applied, resulting in a total of 80 simulations. **Results**: The normalized mean number of spikes in the CA1 region among the virtual AD hippocampi treated with memantine, DBS therapy, and DBS and memantine differed significantly (*p* < 0.0001). The normalized mean number of spikes in the virtual AD hippocampi was 0.33 (95% CI, 0.29–0.36) and was significantly lower compared to the number of spikes in the virtual AD hippocampi treated with memantine, which was 0.53 (95% CI, 0.48–0.59) (*p* = 0.0162), and in the DBS and memantine group, which was 0.67 (95% CI, 0.57–0.78) (*p* = 0.0001). **Conclusions**: Our simulation results indicate the effectiveness of virtual memantine and DBS therapy compared to memantine monotherapy for Alzheimer’s disease.

## 1. Introduction

Alzheimer’s disease (AD) is the most prevalent form of dementia in older adults, characterized by a gradual and progressive decline in cognitive functions, including memory, language, and problem-solving skills. This deterioration significantly hampers the ability to carry out daily activities, placing considerable psychological and social strain on families [1,2]. In later stages, symptoms may extend to disorientation, aggression, depression, and severe long-term memory loss [3].

From a clinical perspective, only a small fraction of AD patients derive therapeutic benefits from pharmacological treatment. Currently, no effective drugs exist to slow down or reverse the progression of AD [4,5,6,7]. The rising incidence and prevalence of AD, a significant health challenge in the aging society of the 21st century, coupled with the limitations of drug therapies, has driven advancements in research on non-pharmacological treatments [8,9] and innovative therapeutic approaches [10]. Both magnetic and electrical stimulation techniques are widely applied in various neurodegenerative diseases [11,12,13,14].

In 1804, Aldini’s application of electrical stimulation marked a new era in the clinical treatment of mental disorders [15,16]. Deep brain stimulation (DBS) is a procedure that surgically implants electrodes into targeted brain structures, using internal generators to deliver controlled electrical currents that modulate brain activity [17,18,19]. Research into DBS as a treatment for AD was initiated after unexpected cognitive improvements were observed in patients receiving DBS for obesity [20] and Parkinson’s disease [21]. DBS is also utilized in the treatment of psychiatric disorders, epilepsy, and depression [13,22,23], as well as a range of neurodegenerative conditions [19,24,25,26]. Research into DBS for AD has shown both positive effects, such as cognitive decline attenuation [9,27,28,29], and controversial negative effects, including postoperative side effects [29,30,31]. Cost-effectiveness studies indicate that DBS is more effective compared to standard clinical treatment for AD patients [32].

Computer modeling [33,34,35,36] and in silico studies [37,38] have effectively contributed to reducing, refining, and partially replacing animal and human experiments. Specifically, the application of gamma oscillation induction in the hippocampus has been shown to alleviate AD-related pathology [39].

The precise mechanism by which DBS affects AD is unknown, and various research hypotheses attempting to explain this phenomenon have yielded inconclusive results [9,40,41,42,43,44]. Most animal studies focus on the behavioral and biological effects of DBS in AD models, including acetylcholine (ACh) release, nerve growth factor (NGF) synthesis induction, and reductions in amyloid-beta (Aβ) and tau protein levels [40,45,46]. However, the majority of human studies have been conducted on small sample sizes, often in conjunction with acetylcholinesterase inhibitors during DBS treatment. Unlike other electrical stimulation methods, DBS is an invasive procedure that carries several risks, such as bleeding, infection, and other complications [47,48,49].

In our study, we aim to conduct a randomized in silico trial comparing virtual DBS therapies with treatment involving an NMDA antagonist, combined with DBS, in AD patients.

## 2. Materials and Methods

### 2.1. The Creation of Biological Multicompartmental Models of the Hippocampus

**Figure 1:**
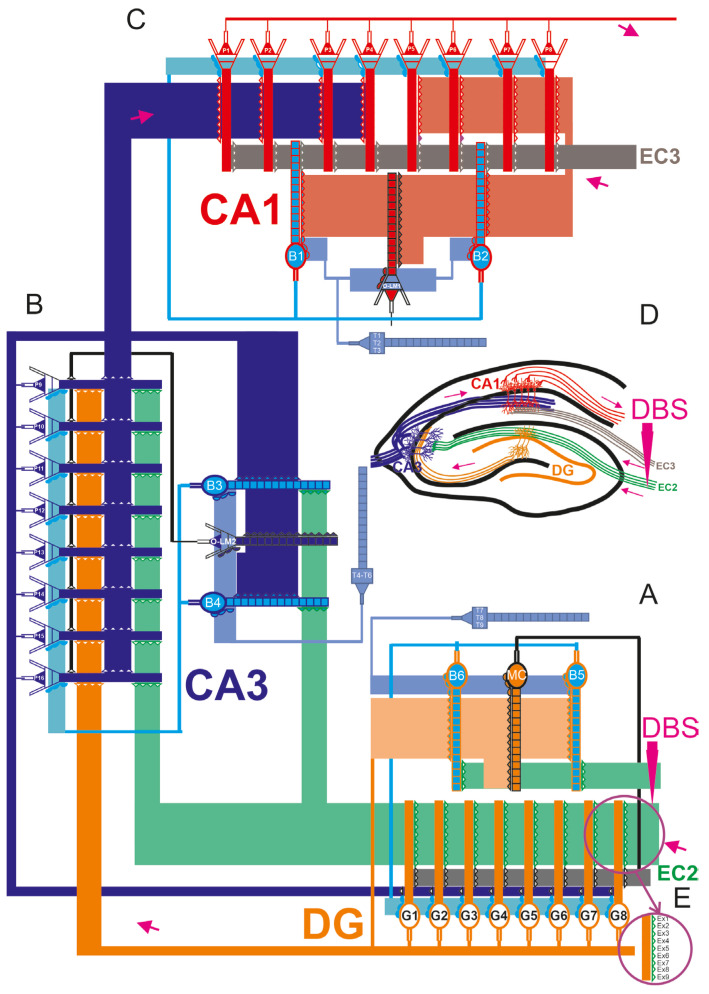
The DG-CA3-CA1 microcircuit of the hippocampal formation, involving the dentate gyrus (DG) region (**A**), CA3 (**B**), CA1 (**C**), and schematic of hippocampal structures including DG, CA3, and CA1: the modeling DBS therapy involved simulating oscillations in the perforant pathway from EC2 to the dentate gyrus and CA3 region, at a frequency of 40 Hz (**D**). Major cell types and their connections include the following: granule cells (G1–G8), Flow of information in the hippocampus from DG through CA3 to CA1, pyramidal cells (CA3 of P1–P16) and (CA1 of P1–P8), basket cells (B1–B6), O-LM1 and O-LM2 cells, mossy cells (MCs), and GABAergic cells (T1–T9) located in the medial septum-diagonal band (MS-DB). These GABAergic cells deliver disinhibitory inputs to hippocampal interneurons operating at theta rhythm. The dentate gyrus and CA3 region receive layer II (EC2) inputs from both the medial and lateral entorhinal cortex, which are radially organized. Meanwhile, principal neurons in the entorhinal cortex layer 3 (EC3) send direct projections to the CA1 field, synapsing with pyramidal neurons in the CA1 region. (**E**) Connections from EC2 to granule cells are shown at Ex1-Ex9, and for Ex1-Ex7, there are bursts of five action potentials (100 Hz) with inter-burst theta frequency at 8 Hz, shifted in phase between particular lines. On lines E × 8 and 9, there were no spikes or silent synapses. For experiments, DBS was added at 40 Hz.

We constructed multicompartmental models of the hippocampus, including granule cells, basket cells, and mossy cells in the DG region. The CA3 and CA1 regions of the hippocampus were composed of pyramidal cells, basket cells, and O-LM cells. Both AMPA and NMDA glutamate receptors, as well as GABA receptors, were incorporated into the network model (Figure 1). The mathematical-computational model of the DG-CA3-CA1 network was based on formalism from previous studies, where the network consisted of 33 neurons. The morphology of nerve cells was simplified to include the cell body, a segment of the axon, and dendrites, incorporating properties detailed in experimental and simulation studies from the literature [34,35,36]. Each cell model consisted of 16 compartments, and had excitatory or inhibitory synapses. Each CA3 pyramidal cell received inhibitory synapses from basket cells and O-LM cells. Excitatory inputs to the CA3 pyramidal cells originated from the entorhinal cortex layer II and the DG. Conversely, basket cells received their stimulating inputs from the distal dendrites of entorhinal cortex layer II and mossy fiber cells in the dentate gyrus. Inputs from EC2 and EC3 were phase-shifted relative to one another, ensuring that strong stimulation from one coincided with weak stimulation from the other. O-LM cells received excitatory inputs from CA3 pyramidal cells and inhibitory signals from the septum. Biological studies indicate that the CA1 region received inputs from mossy fibers, Schaffer collaterals originating in CA3, and projections from the entorhinal cortex layer III. Additionally, theta oscillations, delivered via the septo-hippocampal pathway through the fornix, ranged from 4 Hz to 12 Hz and were temporally coupled with faster gamma oscillations, as simulated in this study. Theta oscillations are thought to play a crucial role in hippocampal functions, particularly in processing spatial information. The network architecture methodology was based on formalism established in prior research [33,34,35,36,39]. The neuronal cell model code is available on the following website: https://github.com/dswietlik/Dariusz-Swietlik/blob/main/Neuron%20model (accessed on 17 February 2022). Computer simulations were performed for the control model and for pathological models (AD).

### 2.2. Study and Treatment Design, Modeling AD and Control

**Figure 2:**
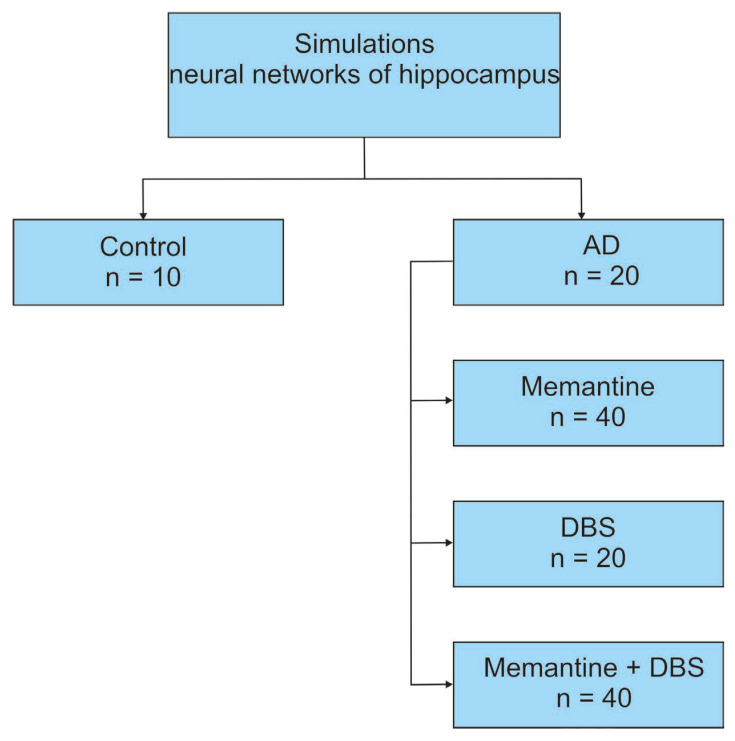
Diagram illustrating the simulation of the hippocampal network. The AD group was randomized to one of three subgroups, where one received virtual memantine therapy, DBS therapy, and memantine and DBS therapy.

The simulation study included the virtual hippocampi of AD patients, represented by neural networks exhibiting moderate-to-severe disease progression. Inclusion criteria encompassed varying degrees of synaptic degradation and levels of excitotoxicity. The modeling of moderate and severe AD was designed to reflect synaptic breakdown in the hippocampus through the loss of synapses. The severity of AD was simulated by progressively disabling connections from the entorhinal cortex layer 2 (EC2) to inhibitory interneurons in the dentate gyrus (DG) granule cells and pyramidal neurons in the CA3 region. Neural networks representing AD patients were randomly assigned (in a 1:2:1 ratio using simple randomization) to the AD group, memantine treatment group, DBS group, and DBS and memantine group. A total of 100 distinct neural networks representing AD patients were generated, with variations in the networks reflecting the modeling of moderate and severe forms of AD, each with different levels of synaptic loss in the hippocampus. The Alzheimer’s disease model was based on the gradual disruption of connections from EC2 to the granule cells of the dentate gyrus, pyramidal neurons in the CA3 region, and inhibitory interneurons, and the control hippocampal network contained no pathology [39]. From these, 20 neural networks were randomly selected to represent AD patients. Subsequently, virtual therapies with memantine, DBS, and DBS and memantine were applied to the selected networks. In total, 100 simulations were conducted (Figure 2).

### 2.3. Virtual Memantine Therapy

The virtual therapy model leveraged the fact that memantine inhibits NMDA receptor currents in a concentration-dependent manner, with IC50 values (the concentration needed for 50% inhibition) ranging from 0.5 to 30 µM at hyperpolarized membrane potentials (−30 to −70 mV). The activation of NMDA receptors and the opening of ion channels are influenced by the synaptic membrane potential. At the resting membrane potential, magnesium ions (Mg^2+^) from the extracellular space enter the channel, blocking its lumen and temporarily preventing the flow of calcium (Ca^2+^) and sodium (Na^+^) ions. However, when postsynaptic receptors are strongly stimulated by glutamate and the overall potential exceeds the NMDA channel opening threshold for calcium ions (−68 mV), the previously blocked channel becomes permeable to Na^+^ and Ca^2+^ ions, allowing them to enter the cell and trigger excitation. In our model, by controlling the NMDA channel opening threshold and recognizing that memantine acts as a voltage-dependent NMDA receptor antagonist, a virtual therapy was conducted at a concentration range of 10 µM.

#### 2.3.1. Normal Synaptic Transmission Model

Normal synaptic transmission involves the activation of receptors by a stimulus, leading to the unblocking of channels and the influx of Ca^2^⁺ ions into the cell. After an action potential arrives, the excitatory synapse input function, InEx, updates the respective shift register tables (E(i): shift register table for AMPA (glutamate) receptors; M(i): shift register table for NMDA (glutamate) receptors). The summarized potential, S(i), determines further activity: if S(i) > CaMT (CaMT = −68 mV, the threshold for removing the Mg^2^⁺ block in NMDA channels), long-term potentiation (LTP) is triggered. Other parameters include C(i): LTP time; ReP: Resting potential (−80 mV); and powerA, clog: additional parameters involved in synaptic regulation.

#### 2.3.2. Neurodegenerative Synaptic Transmission Model

When neurotoxic substances activate receptors, Mg^2^⁺ is displaced, leading to an uncontrolled influx of Ca^2^⁺ ions into the cell. This overactivation of glutamate (excitotoxicity) results in neuronal damage and increased energy demand. The parameter “powerB” (where powerB > powerA) is gradually raised to simulate the rise in extracellular glutamate concentration, which results from excessive NMDA receptor activation and exacerbates excitotoxicity. The following values were used in the control model to represent the intensity of the excitotoxicity effect: 9, 56.7, 63, and 135.

#### 2.3.3. Therapy with Memantine

The depolarization caused by intense stimulation is sufficient to overcome the blockage of the memantine channel, allowing calcium ions to enter the cell. Memantine inhibits NMDA receptor currents in a concentration-dependent manner. Adjustments to the threshold for removing the Mg^2^⁺ ion block from NMDA channels were made to simulate virtual treatment (CaMem > CaMT). The following values were applied in the control model and memantine treatment, respectively: −68, −65, −63, and −55 mV. The approach for this virtual therapy followed the methodology outlined in previous studies [50,51].

### 2.4. Virtual DBS Therapy

DBS modeling involved simulating oscillations in the perforant pathway from entorhinal cortex layer 2 to the DG and CA3 region, using frequencies starting at 40 Hz. The methodology for the modeling DBS is presented in detail in Figure 1E.

### 2.5. Virtual DBS and Memantine Therapy

Virtual DBS and memantine therapy is a simulated therapeutic approach that combines deep brain stimulation (DBS) with the administration of memantine. The simulations employed the integrated mechanisms described in Section 2.3 and Section 2.4.

### 2.6. Measured Parameters

The therapeutic effects were expressed as normalized number of spikes relative to the control model for the output of the hippocampal neural network, specifically in the CA1 region. The secondary endpoints included the number of spikes in the DG and CA3 regions.

### 2.7. Sample Size

Before conducting the virtual simulations of the therapies, calculations for the minimum sample size were performed. The following parameters were adopted: an increase in the number of spikes in the memantine and DBS therapy compared to memantine monotherapy of at least 20%, a population standard deviation common to both sampled populations of no more than 20% relative to the normalized number of spikes, a significance level of alpha = 0.05, and a test power of 80%. The sample size results indicated a total of 20 in each of the virtual therapy groups.

### 2.8. Statistical Analysis

The analyzed parameters of the computer simulations utilized arithmetic means along with 95% confidence intervals. The graphical presentation of the results was illustrated using box-and-whisker plots, incorporating the arithmetic mean, where the box represented the area of mean ± standard error, and the whiskers indicated the 95% CI for the mean. The results of the simulations comparing the AD group treated with memantine, DBS, and DBS and memantine were evaluated using ANOVA, with Tukey’s post hoc test applied in the case of statistical significance. Statistical analysis was conducted using TIBCO Software Inc. (2017). Statistica (data analysis software system), version 13. http://statistica.io. A significance level of α = 0.05 was accepted.

## 3. Results

### 3.1. Normalized Number of Spikes in CA1—The Primary Endpoint

**Figure 3:**
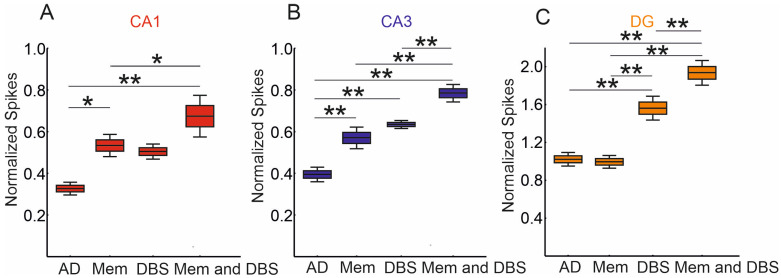
Analysis of simulations of normalized spikes CA1 (**A**), CA3 (**B**), and DG (**C**) (* *p* < 0.05; ** *p* < 0.01). In the box-and-whisker plot, the top and bottom sides of the box are ±1.96 times the variable standard error. The horizontal line that splits the box in two is the mean. The box covers the mean ± 1.96 times the variable standard error.

The normalized mean number of spikes in the CA1 region among the virtual AD hippocampi treated with memantine, DBS therapy, and DBS and memantine differed significantly (*p* < 0.0001). The normalized mean number of spikes in the virtual AD hippocampi was 0.33 (95% CI, 0.29–0.36) and was significantly lower compared to the number of spikes in the virtual AD hippocampi treated with memantine, which was 0.53 (95% CI, 0.48–0.59) (*p* = 0.0162), and in the DBS and memantine group, which was 0.67 (95% CI, 0.57–0.78) (*p* = 0.0001). No statistically significant differences were observed in the normalized mean number of spikes in the virtual AD hippocampus group compared to the DBS therapy group; however, the statistical test result was at the threshold of statistical significance (*p* = 0.0513). Additionally, the number of spikes in the virtual AD hippocampi treated with memantine was significantly lower than the number of spikes in the DBS and memantine therapy (*p* = 0.0223). The difference in the normalized number of spikes between the DBS therapy and DBS and memantine was not statistically significant, but similar to above, the statistical test result was at the threshold of statistical significance (*p* = 0.0679). No statistically significant differences were observed in the normalized mean number of spikes in the virtual AD hippocampi treated with memantine compared to the DBS therapy (*p* = 0.9741) (Figure 3A).

### 3.2. Normalized Number of Spikes in CA3, and DG—The Secondary Endpoints

The normalized mean number of spikes in the CA3 region among the virtual AD hippocampi treated with memantine, DBS therapy, and DBS and memantine differed significantly (*p* < 0.0001). The normalized mean number of spikes in the virtual AD hippocampi was 0.39 (95% CI, 0.36–0.43) and was significantly lower compared to the number of spikes in the virtual AD hippocampi treated with memantine, which was 0.57 (95% CI, 0.52–0.62) (*p* = 0.0004), DBS therapy, which was 0.63 (95% CI, 0.61–0.65) (*p* = 0.0001), and DBS and memantine therapy, which was 0.78 (95% CI, 0.74–0.83) (*p* = 0.0001). Furthermore, the number of spikes in the virtual AD hippocampi treated with memantine was significantly lower than the number of spikes in the DBS and memantine therapy (*p* = 0.0001). No statistically significant differences were observed in the normalized mean number of spikes in the virtual AD hippocampi treated with memantine compared to the DBS therapy group (*p* = 0.4017). A statistically significant increase in the number of spikes was also observed in the DBS and memantine therapy group compared to the DBS therapy group alone (*p* = 0.0023).

The normalized mean number of spikes in the DG region among the virtual AD hippocampi treated with memantine, DBS therapy, and DBS and memantine differed significantly (*p* < 0.0001). The normalized mean number of spikes in the virtual AD hippocampi was 1.02 (95% CI, 0.95–1.10), which was significantly lower compared to the number of spikes in the virtual AD hippocampi treated with DBS therapy, which was 1.56 (95% CI, 1.43–1.70) (*p* = 0.0001), and DBS and memantine therapy, which was 1.93 (95% CI, 1.80–2.07) (*p* = 0.0001). No statistically significant differences were observed in the normalized mean number of spikes in the group of virtual AD hippocampi treated with memantine (*p* = 0.9924). Furthermore, the number of spikes in the virtual AD hippocampi treated with memantine was significantly lower than the number of spikes in the DBS therapy group (*p* = 0.0001) and the DBS and memantine therapy group (*p* = 0.0001). A statistically significant increase in the number of spikes was also observed in the DBS and memantine therapy group compared to the DBS therapy group alone (*p* = 0.0012).

## 4. Discussion

Clinical studies on monotherapy for Alzheimer’s disease have been conducted in the USA, Japan, Austria, the United Kingdom, China, and several other countries, including numerous European nations [52,53,54,55,56,57,58,59,60].

The primary objective of these studies was to evaluate the cognitive function of patients, assessed using scales such as SIB, ADAS, and SMMSE [61]. In five studies, no statistically significant differences were observed compared to placebo, with cognitive function scores for Alzheimer’s patients and control groups ranging from −4.10 to 2.41 and −2.80 to 5.60, respectively [53,54,56,59,62]. However, in four studies, memantine monotherapy was shown to improve cognitive function, with scores ranging from −0.80 to 4.00 compared to placebo (1.10 to 10.10). A meta-analysis of these studies demonstrated a statistically significant improvement in cognitive function, as well as enhancements in daily living activities, global function, and dementia severity. In our in silico study, we confirmed the efficacy of memantine in treating moderate to severe dementia associated with Alzheimer’s disease. The virtual model showed a significant improvement in spike counts within the CA3 and CA1 regions, aligning with clinical trial results that suggest that memantine is effective in treating this condition.

To date, in clinical studies involving patients with Alzheimer’s disease, the DBS targets have included the fornix, the nucleus basalis of Meynert (NBM), and the ventral capsule/ventral striatum (VC/VS) [27,28,29,30,42]. Relevant animal studies have also included various stimulation targets, such as the intralaminar thalamic nucleus (ILN), the midline thalamic nuclei (MTN), the mammillothalamic tract (MT), the anterior nucleus of the thalamus (ANT), the entorhinal cortex (EC), and the CA1 region [63,64]. The entorhinal cortex, a component of the Papez circuit, has been targeted for deep brain stimulation (DBS) to help reduce memory loss [65]. A Canadian research team studied the impact of bilateral DBS of the entorhinal cortex on progressive cognitive decline in a genetic mouse model of Alzheimer’s disease. Their findings showed that high-frequency DBS enhanced hippocampus-dependent memory in young mice 3–6 weeks after stimulation, but no improvement was observed at 1 week post-stimulation. They also showed that deep brain stimulation (DBS) led to a decrease in amyloid plaque accumulation in the hippocampus and cortex of young mice. However, unlike the young mice, older mice did not experience a reduction in plaque burden from DBS, even though their memory function improved successfully. The DBS of the entorhinal cortex (EC) can enhance spatial memory in wild-type mice and promote the rapid proliferation of neurons in the dentate gyrus (DG). The results of the studies were corroborated in our simulation of the dentate gyrus (DG) of the hippocampus, which demonstrated a statistically significant increase in the number of spikes. The stimulation of the entorhinal cortex (EC) using deep brain stimulation (DBS) has been shown to markedly decrease the concentrations of amyloid-beta (Ab) and tau in the hippocampus of transgenic mouse models for Alzheimer’s disease, along with reducing total tau and phosphorylated tau levels in the cortical area [66]. Additionally, another study found that the DBS of the EC can boost synaptic activity by raising synaptophysin levels and facilitate the clearance of tau through the lysosomal pathway, resulting in a positive impact on Alzheimer’s disease [67]. A simulation study evaluated not only the efficacy of memantine monotherapy and deep brain stimulation (DBS) in Alzheimer’s disease but also compared virtual DBS combined with memantine therapy. To the author’s knowledge, no studies to date have assessed the efficacy of combined memantine and DBS therapy in Alzheimer’s disease. However, in 2020, a trial was registered on clinicaltrials.gov titled “The Effect of Deep Brain Stimulation on Memory Network and Neurological Function of Alzheimer’s Disease”, which aims to compare the best medical treatment for Alzheimer’s disease with DBS (NCT03959124). Its results have not yet been published.

Our simulation results indicate a statistically significant increase in the number of spikes in the virtual memantine and DBS therapy group compared to memantine monotherapy in the CA1, CA3, and DG regions of the hippocampus. Furthermore, we observed a statistically significant increase in the number of spikes in these hippocampal regions for the virtual memantine and DBS therapy compared to DBS alone in the CA3 and DG regions of the hippocampus. In the CA1 hippocampal region, no statistically significant improvement was observed in terms of increased spikes for virtual memantine and DBS therapy relative to DBS therapy alone.

## 5. Conclusions

Our simulation results indicate the effectiveness of virtual memantine and DBS therapy compared to memantine monotherapy for Alzheimer’s disease. Moreover, the simulation results in the CA3 and DG regions of the hippocampus also indicate the effectiveness of virtual memantine and DBS therapy compared to DBS therapy alone.

## 6. Limitations

The main limitation of in silico research is the understanding of the capabilities of a mathematical and computational model. The predictions and results of such a model are always shaped by the knowledge applied during its development.

## Data Availability

The data presented in this study are available on request from the corresponding author.
